# Protein secondary structure determines the temporal relationship between folding and disulfide formation

**DOI:** 10.1074/jbc.RA119.011983

**Published:** 2020-01-17

**Authors:** Philip J. Robinson, Shingo Kanemura, Xiaofei Cao, Neil J. Bulleid

**Affiliations:** ‡ Institute of Molecular, Cell, and Systems Biology, College of Medical Veterinary and Life Sciences, Davidson Building, University of Glasgow, Glasgow G12 8QQ, United Kingdom; § Kwansei Gakuin University, 2-1 Gakuen, Sanda, Hyogo 669-1337, Japan

**Keywords:** disulfide, endoplasmic reticulum (ER), protein disulfide isomerase, protein secretion, protein folding, protein structure, folded precursor model, nascent chain, protein homeostasis, protein translocation, quasi-stochastic model

## Abstract

How and when disulfide bonds form in proteins relative to the stage of their folding is a fundamental question in cell biology. Two models describe this relationship: the folded precursor model, in which a nascent structure forms before disulfides do, and the quasi-stochastic model, where disulfides form prior to folding. Here we investigated oxidative folding of three structurally diverse substrates, β2-microglobulin, prolactin, and the disintegrin domain of ADAM metallopeptidase domain 10 (ADAM10), to understand how these mechanisms apply in a cellular context. We used a eukaryotic cell-free translation system in which we could identify disulfide isomers in stalled translation intermediates to characterize the timing of disulfide formation relative to translocation into the endoplasmic reticulum and the presence of non-native disulfides. Our results indicate that in a domain lacking secondary structure, disulfides form before conformational folding through a process prone to nonnative disulfide formation, whereas in proteins with defined secondary structure, native disulfide formation occurs after partial folding. These findings reveal that the nascent protein structure promotes correct disulfide formation during cotranslational folding.

## Introduction

A central question in protein folding is how proteins that contain multiple cysteines achieve the correct disulfide pattern. Intramolecular disulfide bonds consist of covalent cross-links between cysteines within a polypeptide (1), and their formation imposes conformational restraints that influence protein folding, stability, and function. Disulfides form through an oxidative reaction between paired cysteines in the presence of an appropriate catalyst (2). In eukaryotic cells, the endoplasmic reticulum (ER)^3^ is the main site for disulfide formation; it provides a favorable redox environment and catalysts in the form of resident protein disulfide isomerase (PDI) family members (3). When polypeptides contain more than two cysteines, correct pairing is required to form the native disulfide pattern; the spatial positioning of cysteine side chains is required for this correct pairing, closely linking disulfide formation to the process of conformational folding.

Two models have been proposed that broadly explain the relationship between disulfide formation and conformational folding: the folded precursor mechanism, in which nascent structure forms first, which then positions paired cysteines favorably to form a disulfide, and the quasi-stochastic model, in which cysteines in an unfolded precursor pair more randomly, and the resulting disulfide influences further folding (4). Recent studies have identified substrates that favor the folded precursor mechanism (5–7), whereas other studies have identified contributions from both mechanisms at different stages of the folding process (8). Incorrect (non-native) disulfides can also form during folding and are subsequently reduced and rearranged (9, 10). In the ER, this reduction step is performed by specific PDI family members (11–13) using reducing equivalents that originate in the cytosol (14). Evidence shows that non-native disulfides can act as important intermediates in the native folding of certain proteins (10).

Folding and disulfide formation in the cell is a vectorial process. During the early stages of translation, nascent polypeptides enter the ER through the Sec translocon (15). The space constraints of the ribosome–Sec complex limit structure formation until enough polypeptide has entered the ER lumen (16). Addition of each amino acid to the polypeptide expands the repertoire of intramolecular interactions and the diversity of potential folds that might form. Both native and non-native disulfides can form at this cotranslational stage (17, 18). Exposure of the nascent polypeptide to the ER as well as folding of the nascent polypeptide and the accessibility of PDI family members dictate native disulfide formation (19). Non-native disulfides may also form because of the partial exposure of nascent polypeptides to the ER, which prevents completion of folding, leading to incorrect cysteine coupling.

The question of how proteins with multiple cysteines achieve the correct disulfide pattern is difficult to address because of the structural diversity of disulfide-containing proteins and the technical barriers that hinder folding assays performed in a biologically relevant context. In an earlier study, we devised an approach to investigate the folding of a protein containing a single disulfide as it emerges into the ER lumen (7). Here we expand this approach to study nascent polypeptides with diverse structures and complex disulfide patterns to investigate the relationship between protein folding and correct disulfide formation. Our results demonstrate the influence of protein secondary structure on the fidelity of disulfide formation in a cellular context.

## Results

### Experimental approach

**Figure 1.**
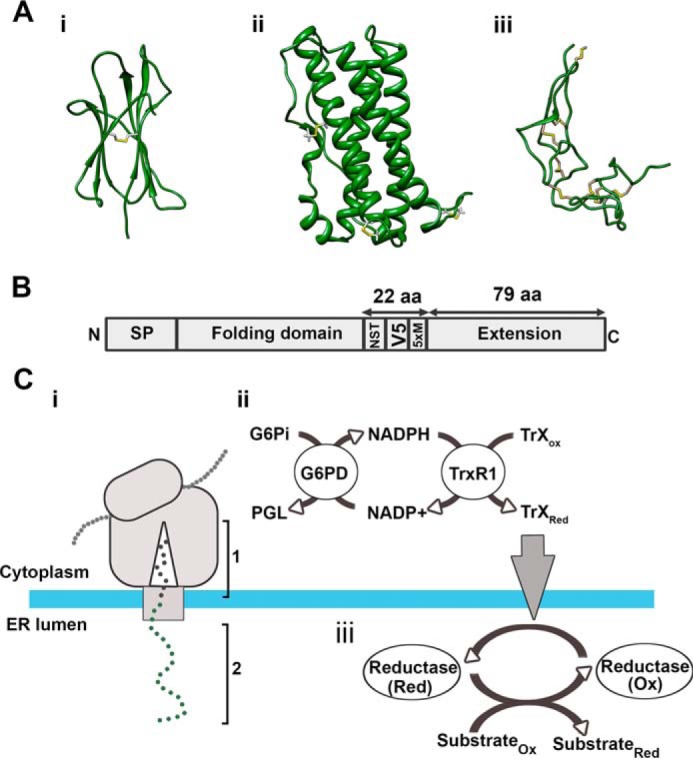
**An experimental system to monitor disulfide formation and rearrangement in translation intermediates.** *A*, ribbon diagrams representing the substrates used in this study: *i*, human β2M (PDB code 1A1M); *ii*, human prolactin (PDB code 1RW5); *iii*, the disintegrin domain of human ADAM10 (PDB code 6BE6). Disulfide bonds are highlighted in *yellow. B*, schematic illustrating the C-terminal extension added to each folding domain to produce the extended constructs. The position of the signal peptide, glycosylation site (NST), V5 epitope, and five methionine residues (*5*×*M*) are indicated. *C*, schematic of the ribosome–Sec complex expressing an ER-exposed nascent polypeptide (*i*). Phase 1 shows how the C-terminal extension retains the polypeptide attached to the ribosome to allow phase 2. In phase 2, the N-terminal folding domain of the polypeptide is fully exposed to the ER lumen. G6Pi drives the cytosolic reducing pathway (*ii*), which is the source of reducing equivalents for disulfide rearrangements in the ER (*iii*). *PGL*, phosphoglucolactone; *G6PDH*, glucose 6-phosphate dehydrogenase; *TrxR1*, thioredoxin reductase; *TrX*, thioredoxin.

The three proteins used in this study, β2-microglobulin (β2M), prolactin, and the disintegrin domain of ADAM10, were chosen for their diversity of secondary structure and disulfide bonding (Fig. 1*A*). We added a C-terminal extension to each protein (Fig. 1*B*) to act as a tether to the ribosome and to enable the folding domain to enter the ER lumen before completion of translation (Fig. 1*C*, *i*). Between the extension and the folding domain, we engineered a glycosylation site (NST) to monitor translocation, a V5 epitope tag for immunoisolation, and five methionine residues to boost the signal of the radiolabel. DNA templates were synthesized that encode specific polypeptide lengths that lack stop codons. The templates were transcribed, and the resulting mRNA was used to program translation reactions to produce stalled ribosome–nascent chain complexes (RNCs). Translations were performed using a rabbit reticulocyte lysate supplemented with semipermeabilized (SP) cells to study folding in the ER (20) or without SP cells to represent folding in the cytosol. All samples were treated with NEM upon completion to irreversibly modify thiols and freeze the disulfide status of the samples for downstream processing.

We adjusted the redox status of the translation reactions by adding specific components to the rabbit reticulocyte lysate. In all, we used three different lysates in our experiments: a reducing lysate supplemented with DTT, in which proteins synthesized are unable to form disulfides in the absence or presence of SP cells; an oxidizing lysate that contained no additional components (this lysate allows disulfides to form in proteins synthesized in the absence or presence of SP cells); and a redox-balanced lysate supplemented with G6Pi to drive glucose 6-phosphate-dehydrogenase and TrxR1 activity (14) (Fig. 1*C*, *ii*). This lysate is sufficiently reducing to prevent disulfide formation in proteins synthesized without SP cells but allows disulfide formation in translocated proteins when SP cells are present (14). Proteins synthesized in the redox-balanced lysate supplemented with SP cells have the capacity for disulfide reduction and rearrangements. This is because the activity of cytosolic glucose-6-phosphate dehydrogenase and TrxR1 is the source of reducing equivalents for an ER-reductive pathway, which is required to reduce non-native disulfides (Fig. 1*C*, *iii*). Thus, by comparing disulfide formation in oxidizing and redox-balanced lysates, we can assess the influence of the reducing pathway on the fidelity of disulfide formation.

Throughout this paper, we use the term “folding” to reflect the process during which proteins fold to form partially structured states on their way to the native tertiary structure. Hence, if a protein is folding, it has not necessarily attained its native structure. We refer to proteins that have adopted their native structure as “folded.”

### Stochastic disulfide formation is absent in β2M

**Figure 2.**
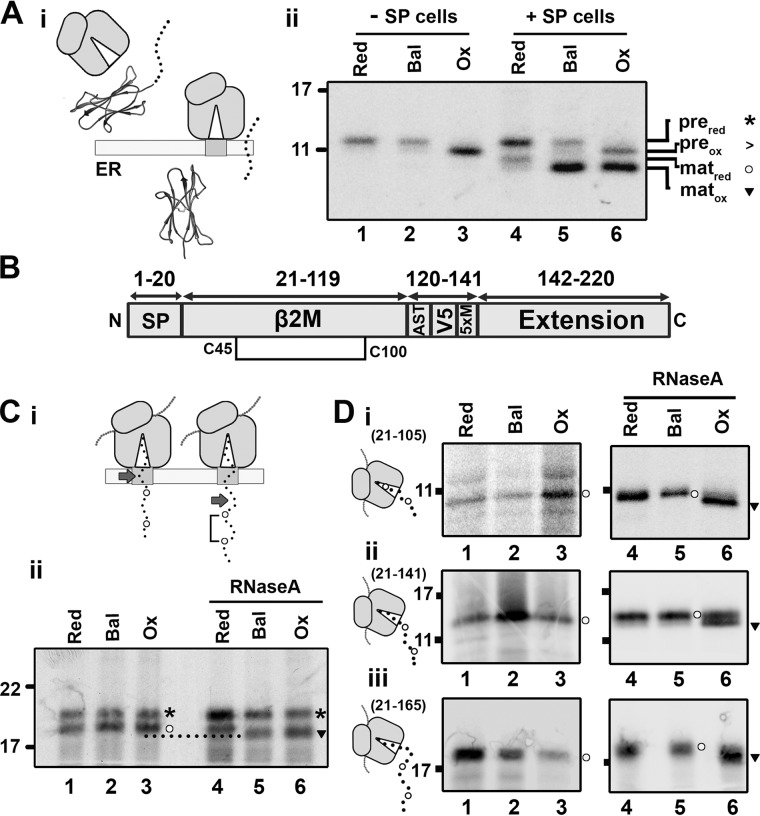
**β2M-disulfide formation is folding-driven.** *A*, *i*, diagram illustrating signal peptide cleavage, which occurs following translocation of β2M. *ii*, nonreducing SDS-PAGE of immunoisolated WT β2M translated with or without SP cells in reducing (*Red*), redox-balanced (*Bal*), or oxidizing (*Ox*) lysate. The migration of preprotein (*pre*) and mature protein (*mat*) when oxidized (*ox*) or reduced (*red*) is indicated by the associated symbols (*asterisks*, >, *circles*, and *inverted triangles*). *B*, organization of the extended β2M construct with the single disulfide (Cys^45^-Cys^100^) displayed. *C*, *i*, diagram illustrating the dependence of disulfide formation on domain exposure; *arrows* highlight the end of the β2M folding domain. *ii*, nonreducing SDS-PAGE shows isolated 175 intermediates translated with SP cells in redox-balanced, oxidizing, and reducing lysate. RNaseA treatment following translation is indicated. *D*, nonreducing SDS-PAGE analysis of isolated ribosome/nascent chain complexes representing β2M 21–105 (*i*), 21–141 (*ii*), and 21–165 (*iii*) translated in redox-balanced, oxidizing, or reducing lysate without SP cells. Samples treated with RNaseA following translation are indicated. Schematics next to each panel indicate the predicted cysteine exposure at each intermediate length. All gels in this figure are representative of at least three repeats.

β2M is a small, β-sheet–rich protein with two cysteines and a single disulfide (Fig. 1*A*, *i*). It has an N-terminal signal peptide that is cleaved following ER targeting (Fig. 2*A*, *i*). We can follow disulfide formation in β2M by non-reducing SDS-PAGE, as it has faster mobility than the reduced protein (7). When WT β2M was translated without SP cells in the redox-balanced or reducing lysates, the resulting product migrated as a single species corresponding to the reduced preprotein (Fig. 2*A*, *ii*, *lanes 1* and *2*; *pre_red_*). When translated in the oxidizing lysate, a faster migrating species was detected, indicating disulfide formation (Fig. 2*A*, *ii*, *lane 3*; *pre_ox_*). When β2M was synthesized in the presence of SP cells, ER targeting resulted in signal peptide cleavage with the translation product from the reducing lysate migrating as two species representing reduced preprotein (pre_red_) and reduced mature protein (mat_red_) (Fig. 2*A*, *ii*, *lane 4*). The mature protein from the redox-balanced and oxidized lysates runs faster (mat_ox_) relative to mat_red_, indicating disulfide formation. Disulfide formation also occurred in preprotein translated in the oxidizing lysate (Fig. 2*A*, *ii*, *lane 6*). These results demonstrate the capacity of the different lysates for disulfide formation and how this can be detected for β2M on non-reducing SDS-PAGE.

In a previous study, we used an extended β2M sequence (Fig. 2*B*) to investigate folding events during ER entry and found that disulfide formation is a folding-driven process that requires the β2M sequence to be fully translocated into the ER (Fig. 2*C*, *i*) (7). It was also found in our previous work that PDI binds throughout the translocation process and is likely to catalyze disulfide formation at a late stage of folding. These findings are consistent with a structured precursor mechanism of disulfide formation. We performed these previous experiments in a redox-balanced lysate, and, consequently, the ER-reductive pathway may prevent or correct any stochastic disulfide formation that occurs in unstructured or partially folded intermediates prior to full translocation of the folding domain. To investigate this, we translated the 175 intermediate of extended β2M in the absence (oxidizing lysate) or presence (redox-balanced lysate) of a robust ER-reductive pathway (Fig. 2*C*, *ii*). At this intermediate length, both cysteine residues will have exited the Sec complex, whereas part of the folding domain remains within. The RNA transcript did not contain a stop codon, preventing its release from the ribosome. The resulting translation products had the same migration independent of the lysate used (Fig. 2*C*, *ii*, *lanes 1–3*), indicating lack of any disulfide formation when the nascent chain is tethered to the ribosome. For the RNase-treated samples (Fig. 2*C*, *ii*, *lanes 4–6*), disulfide formation was absent from preprotein under all conditions. This result contrasts with formation of a disulfide in the mature protein when translated in the presence of a stop codon (Fig. 2*A*, *ii*, *lane 6*). This may reflect lack of a stalled nascent chain. In contrast, the mature protein forms disulfides when translated in both oxidizing and redox-balanced lysates (Fig. 2*C*, *ii*, *lanes 5* and *6*, *inverted triangle*). This result demonstrates that the absence of disulfide formation prior to full translocation of the β2M folding domain is independent of the ER-reductive pathway. This indicates that folding via the structured precursor mechanism controls the timing of disulfide formation under these conditions and negates the need for reductive mechanisms to prevent premature disulfide formation.

To investigate the role of ER factors in folding and disulfide formation, we synthesized stalled RNCs of β2M in oxidizing lysate in the absence of SP cells. We showed previously that these conditions are sufficient to induce disulfide formation in WT β2M released from the ribosome (Fig. 2*A*, *ii*). Polypeptide synthesis in the absence of ER factors to regulate disulfide formation may favor stochastic disulfide formation. For these experiments, we used a β2M sequence that did not contain an N-terminal signal sequence to represent the mature protein in case the presence of the signal peptide affected folding. Three intermediate lengths were chosen based on estimates assuming that ∼40 amino acids span the length of the ribosome tunnel (two-thirds of the amino acids required to span the entire ribosome–Sec complex) (7, 21). The schematics in Fig. 2*D* illustrate the predicted cysteine exposure in each case. Following translation in the different lysates, stalled RNCs were either isolated by ultracentrifugation through a sucrose cushion or immunoisolated following RNaseA treatment (Fig. 2*D*, *i–iii*). No disulfide formation occurred for stalled and released intermediates translated in redox-balanced or reducing lysates (Fig. 2*D*, *i–iii*, *lanes 1*, *2*, *4*, and *5*). The stalled intermediates translated in the oxidizing lysate showed no evidence of disulfide formation either (Fig. 2*D*, *i–iii*, *lane 3*), despite exposure of both cysteines to the oxidizing lysate in the 141 and 165 intermediates. Disulfide formation occurred only following release of the nascent chains from the ribosome (Fig. 2*D*, *i–iii*, *lane 6*).

These results with β2M support our earlier findings (7) suggesting that disulfide formation requires a folding-driven mechanism. Here we extended this conclusion to include conditions where stochastic disulfide formation is more likely because of the lack of influencing ER folding factors and reducing pathways. The translations in oxidizing lysate both with and without SP cells show that the entire folding domain needs to exit the ribosome before disulfide formation can take place. This suggests that, even under these conditions, disulfides form through a controlled process driven by folding rather than a stochastic process in which premature disulfides form.

### Disulfide formation in prolactin favors a structured precursor folding model

**Figure 3.**
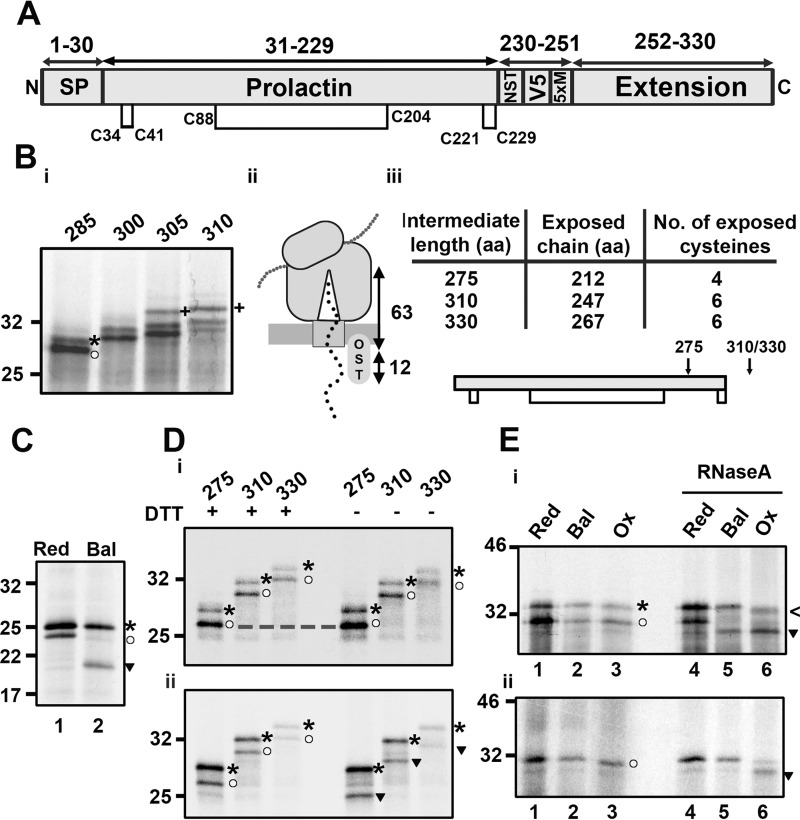
**Disulfide formation in prolactin only occurs after ribosome release.** *A*, organization of the extended prolactin construct with disulfide bond positions indicated. *B*, *i*, reducing SDS-PAGE of extended prolactin intermediates (285–310 aa in length), translated with SP cells. Preprotein (*asterisk*) and mature protein (*circle*) are highlighted for the 285 intermediate, and glycosylated protein is highlighted for the 305 and 310 intermediates (+). *ii*, schematic of the extension length (63 aa) required for ER exposure. *iii*, table of prolactin intermediate lengths containing the predicted exposure of amino acids and cysteine residues to the ER lumen and a topology diagram with ER exposure of specific intermediates, indicated by *arrows. C*, nonreducing SDS-PAGE of WT prolactin translated with SP cells in reducing (*Red*) or redox-balanced (*Bal*) lysate. D, extended prolactin intermediates (275–330) lacking a glycosylation site were translated with SP cells in redox-balanced lysate and either stalled (*i*) or released through RNaseA treatment (*ii*). Samples were immunoisolated (*V5*) and run under reducing (+*DTT*) and non-reducing (−*DTT*) conditions. *E*, non-reducing SDS-PAGE of the 310 intermediate translated in reducing, redox-balanced, or oxidizing (*Ox*) lysate in the presence (*i*) or absence (*ii*) of SP cells. Stalled samples were compared with released (RNaseA-treated) samples. In *C–E*, bands corresponding to reduced preprotein (*asterisks*), reduced mature protein (*circles*), oxidized preprotein (<), and oxidized mature protein (*inverted triangles*) are highlighted. Representative data from at least three experimental repeats in each case are shown.

The next substrate studied was bovine prolactin, which has an α-helical structure and three native disulfide bonds (22). The schematic in Fig. 3*A* illustrates the extended-prolactin construct. We monitored *N*-linked glycosylation at position 230 to determine the intermediate length required for prolactin domain exposure to the ER lumen (Fig. 3*B*). Following translation of increasing lengths of the extended prolactin, glycosylation occurred from an intermediate length of 305 amino acids (Fig. 3*B*, *i*). We calculated that 75 amino acids (305–230) are required to span the distance between the P-site of the ribosome and the active site of the oligosaccharyl transferase. Taking into account the ∼12 residues it takes to reach from the exit of the Sec complex to the oligosaccharyl transferase, we estimate that 63 residues span the ribosome–Sec complex (Fig. 3*B*, *ii*), the same value observed with the β2M construct (7). Based on this result, we predicted the length of polypeptide exposed to the ER for each intermediate, as shown in the table and topology diagram in Fig. 3*B*, *iii*. For the remaining experiments with prolactin, we used a construct without the glycosylation site to simplify SDS-PAGE analysis.

The presence of the long-range disulfide (Cys^88^-Cys^204^) in prolactin polypeptides results in a significant mobility difference when comparing reduced and non-reduced samples on SDS-PAGE (Fig. 3*C*). The long-range disulfide forms in the WT protein when synthesized in the redox-balanced lysate (Fig. 3*C*, *lane 2*). The two short-range disulfides do not influence SDS-PAGE mobility and so are not detected in these assays. As prolactin contains six cysteines, there is also the potential for long-range, non-native disulfides to form that may also influence gel mobility. To determine whether long-range disulfides form in prolactin nascent chains, we translated a range of stalled intermediates in redox-balanced lysate and compared the resulting gel mobility under reducing (+DTT) or non-reducing (−DTT) conditions (Fig. 3*D*, *i*). Of the three intermediate lengths tested (275, 310, and 330), the migration of the signal peptide–cleaved polypeptide is the same under non-reducing or reducing gel conditions, revealing an absence of disulfide formation in each case. In contrast, samples treated with RNaseA to release nascent chains from the RNCs formed disulfides (Fig. 3*D*, *ii*). Based on the glycosylation results (Fig. 3*B*, *iii*), the 310 and 330 RNCs have a prolactin domain exposed to the ER, and yet the long-range disulfide fails to form until nascent chain release from the ribosome.

We tested whether the ER-reductive pathway is responsible for preventing disulfide formation in the RNCs by synthesizing prolactin in the presence of SP cells in oxidizing lysate. Translation of the 310 intermediate resulted in a polypeptide with the same gel mobility as that produced from translation in redox-balanced or reducing lysates (Fig. 3*E*, *i*, *lanes 1–3*). Disulfide formation was observed only following release of the nascent chain (Fig. 3*E*, *i*, *lanes 5* and *6*). This result demonstrates that the ER-reductive pathway is not actively reducing prolactin intermediates to prevent premature disulfide formation. We also tested whether disulfides could form in the absence of added SP cells (Fig. 3*E*, *ii*). For this assay, we translated a prolactin construct that did not contain the N-terminal signal sequence to represent the mature protein (residues 31–310) and was of sufficient length (280 aa) to ensure that the folding domain exited the ribosomal tunnel. When this intermediate was translated in the oxidizing lysate, SDS-PAGE analysis showed an absence of disulfide formation in the RNCs (Fig. 3*E*, *ii*, *lane 3*) before RNaseA-induced release (Fig. 3*E*, *ii*, *lane 6*). Polypeptides synthesized in the redox-balanced and reducing lysates showed no disulfide formation in either stalled or released polypeptides. These results demonstrate that the requirement for nascent chain release before disulfide formation is independent of ER factors.

Mechanistically, the absence of both native and non-native disulfide formation upon cysteine exposure to the oxidizing milieu shows that stochastic disulfide formation is absent. This indicates that disulfide formation does not occur via the quasi-stochastic mechanism of folding, in which disulfides form first in unstructured, folding precursors before conformational folding takes place. Instead, ribosome-attached intermediates are likely to form stable structures that separate cysteines and actively prevent disulfide formation at this stage. Only upon release is the conformational freedom necessary for correct folding achieved, which then positions cysteines accurately for native disulfide formation. These results thus favor the structured precursor model of oxidative folding.

### Multiple disulfide species in partially exposed disintegrin intermediates indicate a stochastic mechanism of disulfide formation

**Figure 4.**
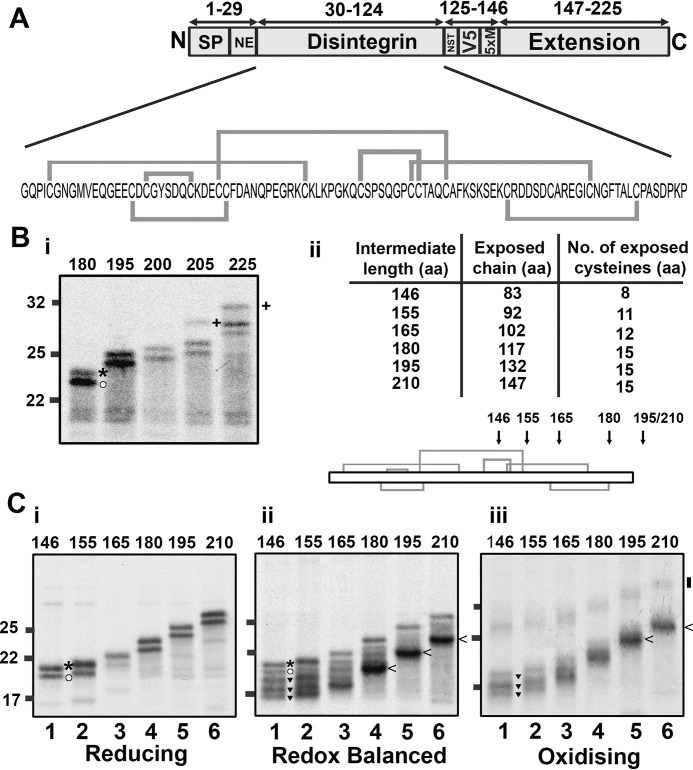
**Disulfide formation occurs in a partially ER-exposed disintegrin domain.** *A*, organization of the disintegrin construct with the sequence and native disulfide bonding pattern highlighted. *B*, *i*, reducing SDS-PAGE to identify glycosylation (+) in extended disintegrin intermediates of lengths of 180–225 (preprotein (*asterisk*) and mature protein (*circle*) are highlighted for the 180 intermediate) (*i*), with the table and topology diagram (*ii*) detailing expected exposure of amino acids and cysteine residues to the ER lumen. *C*, non-reducing SDS-PAGE showing radiolabeled intermediates of increasing length (146–210 preprotein length) translated in reducing (*i*), redox-balanced (*ii*), and oxidizing lysate (*iii*). For the 146 samples, gel bands representing reduced preprotein (*asterisks*), reduced mature protein (*circles*) and oxidized mature protein (*inverted triangles*) are indicated. All gels represent translation reactions with SP cells immunoisolated to the V5 epitope and are representative of three independent repeats.

β2M and prolactin have stable secondary structures in which the disulfide density is low; these features may favor the structured precursor model of oxidative folding. To investigate whether a contrasting protein structure folds via an alternative mechanism, we studied the disintegrin domain of ADAM10. This protein domain has a dense disulfide bonding pattern and little defined secondary structure (Fig. 1*A*, *iii*) (23, 24). The importance of the disulfide bonds in defining the structure makes it more likely that they form earlier in the folding process, perhaps in unfolded precursors. We illustrate the organization of the disintegrin construct and its pattern of disulfide bonding in Fig. 4*A*. We monitored glycosylation at position 125 to determine the intermediate length required for disintegrin domain exposure to the ER lumen (Fig. 4*B*, *i*) using the same procedure as described for the prolactin construct. Glycosylation occurred from a length of 200 amino acids onward, which corresponds to the 63 amino acids of extension sequence required to span the length of the ribosome/Sec channel. This is the same length observed for β2M (7) and prolactin (Fig. 3*B*). We depict the predicted ER exposure, in terms of polypeptide length and number of cysteines exposed to the ER, in Fig. 4*B*, *ii*, with *arrows* in the topology diagram illustrating ER exposure expected at specific intermediate lengths. For the remaining experiments with disintegrin, we used a construct without the glycosylation site to simplify SDS-PAGE analysis.

To evaluate disulfide bonding in the disintegrin domain relative to translocation, we expressed a range of intermediates with increasing levels of ER exposure (Fig. 4*C*). When translated in the presence of SP cells and in a reducing lysate, we observed two products in each lane that corresponded to reduced preprotein and reduced mature protein (Fig. 4*C*, *i*). When translated in a redox-balanced lysate, the shorter intermediates ran as five distinct species (Fig. 4*C*, *ii*, *lanes 1–3*). By comparing these with the reduced sample, we concluded that the slowest-migrating species corresponds to untranslocated, reduced preprotein and that the four faster migrating species represent translocated species with different disulfide bond configurations. The clear separation of the species indicates that the disulfides produce specific long-range constraints that contribute to changes in SDS-PAGE mobility. This early disulfide formation and the mixed population of species that result indicate a quasi-stochastic mechanism of cysteine coupling. For the longer intermediates, the abundance of each gel species changes, indicating that the disulfide configuration that forms is highly dependent on the degree of ER exposure (Fig. 4*C*, *ii*, *lanes 4–6*). We observed a distinct faster-migrating species when the disintegrin domain was fully exposed (Fig. 4*C*, *ii*, *lanes 4–6*, <). This demonstrates that a single disulfide pattern is favored when the entire disintegrin domain enters the ER lumen.

When we performed the same translation reactions in an oxidizing lysate, a distinctly different gel migration pattern was observed (Fig. 4*C*, *iii*). Under these conditions, disulfide formation can occur in untargeted preprotein, and the ER loses the capacity for disulfide rearrangement. The absence of products that correspond to the reduced protein shows that disulfide formation is extensive. For each sample, the fast-migrating gel species correspond to intermediates with intrachain disulfides (Fig. 4*C*, *iii*, < for the 210 intermediate), and the slower migrating species indicates the presence of interchain disulfides (Fig. 4*C*, *iii*, *vertical bar* for the 210 intermediate). The intrachain species that formed in the oxidizing lysate showed more diffuse patterns on SDS-PAGE compared with those synthesized in the redox-balanced lysate, indicating the presence of a heterogeneous mixture of disulfide-bonded species. As the intermediates increased in length, a less heterogeneous mix of species became evident (Fig. 4*C*, *iii*, *lanes 5* and *6*). These results indicate that the disintegrin disulfides formed in the oxidizing lysate are slow to rearrange into distinct intermediates, resulting in an ensemble of disulfide-bonded species.

### ER-specific disulfide rearrangements occur in disintegrin nascent chains

The disulfides formed in partially exposed disintegrin intermediates are likely to be non-native disulfides that require subsequent rearrangement. Disulfide rearrangement mechanisms depend on the ER-reductive pathway. The activity of this pathway could explain why we see a different disulfide pattern when comparing partially exposed intermediates translated in redox-balanced and oxidizing lysates. In the above experiments with the disintegrin domain, the translation products were immunoisolated using the V5 antibody, which recognizes the V5 epitope positioned between the folding domain and extension (Fig. 4*A*), to isolate both preprotein and mature protein. Judging by the intensity of bands representing preprotein and mature protein under reducing conditions (Fig. 4*C*, *i*), only about 50% of the protein is successfully translocated and cleaved. This mixed population makes it difficult to define which of the multiple bands observed under redox-balanced and oxidizing conditions originate from translocated polypeptides and which, if any, originate from disulfide formation in the lysate. To define disulfide formation and rearrangements taking place in ER-translocated intermediates requires a method to separate preprotein and mature protein. For this purpose, we utilized a neo-epitope (NE) tag placed between the signal peptide and the disintegrin domain. This epitope is only recognized when the signal peptide is cleaved, allowing isolation of mature protein without contamination from preprotein (25).

**Figure 5.**
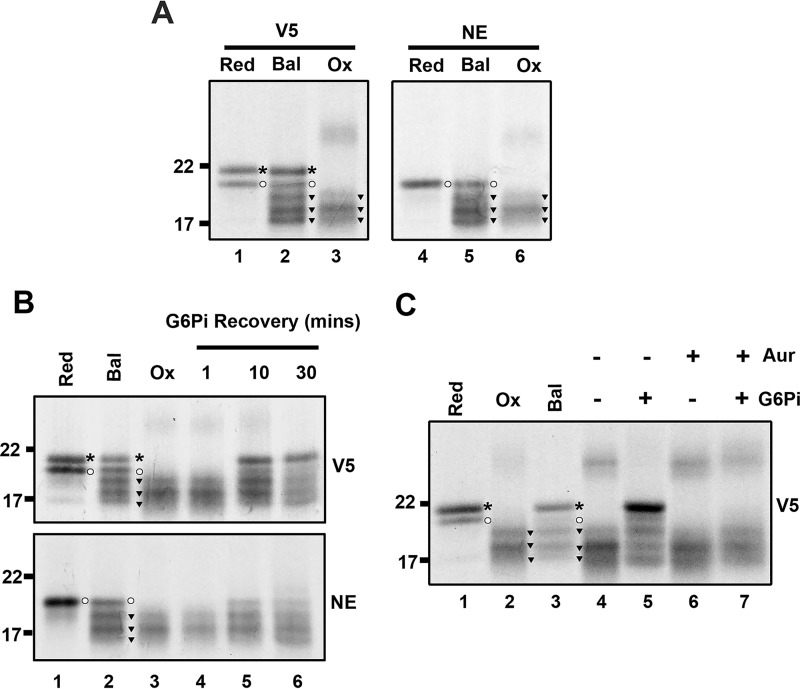
**ER-specific disulfide rearrangements occur in a partially ER-exposed disintegrin intermediate.** *A–C*, non-reducing SDS-PAGE shows either total translation product (V5) or ER-specific species (NE) for the 146 intermediate of the disintegrin domain when translated in reducing (*Red*), redox-balanced (*Bal*), and oxidizing (*Ox*) lysate (A); when translated in oxidizing lysate with G6Pi added after translation and samples taken at specific time points after G6Pi treatment (*B*, *lanes 4–6*); and when translated in oxidizing lysate to which G6Pi was added after translation in the presence or absence of auranofin (*Aur*) (*C*, *lanes 4–7*). Control samples for *B* and *C* were translated in reducing, redox-balanced, and oxidizing lysates (*lanes 1–3* in both cases) for gel mobility comparison. The experiments in *A* and B were repeated three times and those in *C* twice, with representative data shown. Symbols indicate reduced preprotein (*asterisks*), reduced mature protein (*circles*), and oxidized mature protein (*inverted triangles*).

To identify ER-specific disulfide bonded species, we translated the intermediate length 146 with SP cells under reducing, redox-balanced, or oxidizing lysates (Fig. 5*A*). Samples were immunoisolated using the NE or V5 antibody and separated under non-reducing conditions. Following V5 immunoisolation, translation products from the reducing lysate migrated as two species corresponding to preprotein and mature protein (Fig. 5*A*, *lane 1*) and a single band corresponding to mature protein following NE isolation (Fig. 5*A*, *lane 4*). This confirmed that the NE antibody only recognizes mature protein. The results with redox-balanced lysate showed that, of the five species detected following V5 isolation (Fig. 5*A*, *lane 2*), all except the reduced preprotein were ER-translocated species (Fig. 5*A*, *lane 5*). Analysis of the same intermediate translated in the oxidizing lysate showed the fast-migrating smear in the V5-isolated sample (Fig. 5*A*, *lane 3*) and the NE-isolated sample (Fig. 5*A*, *lane 6*), confirming that these products also represent ER-translocated, disulfide-bonded species. These results show that non-native disulfides form in oxidizing lysate and that the difference in disulfide bonding compared with the redox-balanced lysates is due to the activity of the ER-reductive pathway. Formation of non-native disulfides in the disintegrin domain indicates that cysteines are pairing through a quasi-stochastic mechanism.

Having confirmed that non-native disulfides can form in partially translocated intermediates, we next investigated whether we could directly monitor non-native disulfide rearrangements in nascent chains. To do so, we synthesized the 146 intermediate in oxidizing lysate and added G6Pi after nascent chain synthesis to activate the reductive pathway posttranslationally (Fig. 5*B*). Translation products synthesized in reducing, redox-balanced, or oxidizing lysate were run alongside the samples for comparison (Fig. 5*B*, *lanes 1–3*). Over the 30-min time course following G6Pi addition, the mobility of the translation products changed from a diffuse smear (Fig. 5*B*, *lane 4*, *1 min*) to the equivalent pattern formed in redox-balanced lysate (Fig. 5*B*, *lanes 5* and *6*, *10–30 min*). The NE-isolated samples showed the same pattern as the V5-isolated samples but without reduced preprotein. These results demonstrate that disulfide rearrangements can take place in nascent chains prior to release from the ribosome.

Finally, we confirmed that thioredoxin reductase is the source of reducing equivalents for the disulfide rearrangements observed by performing G6Pi recovery experiments in the presence of the thioredoxin reductase inhibitor auranofin. Treatment with auranofin prevented disulfide rearrangement (Fig. 5*C*, compare *lanes 5* and *7*), which confirms that cytosolic thioredoxin reductase is the source of reducing equivalents for these disulfide rearrangements.

### Decreasing cysteine density of the disintegrin domain does not prevent stochastic disulfide formation

The disintegrin domain has a higher density of disulfide bonds than the substrates that follow the structured precursor mechanism. High cysteine density could be a feature that favors quasi-stochastic coupling. To test whether lowering the cysteine density alters the folding mechanism, we generated disintegrin constructs with specific cysteine residues substituted for serine. We designed three constructs with four, three, or one native cysteine pairs, termed the N-term cluster (four native disulfides located at the N terminus), triple (three sequential disulfides), and single (one long-range disulfide). Intermediates were produced that either enabled partial (146 or 165) or full (210) translocation of the disintegrin domain to the ER. Following translation, translation products were immunoisolated using V5 and NE antibodies to compare total and ER-translocated products.

**Figure 6.**
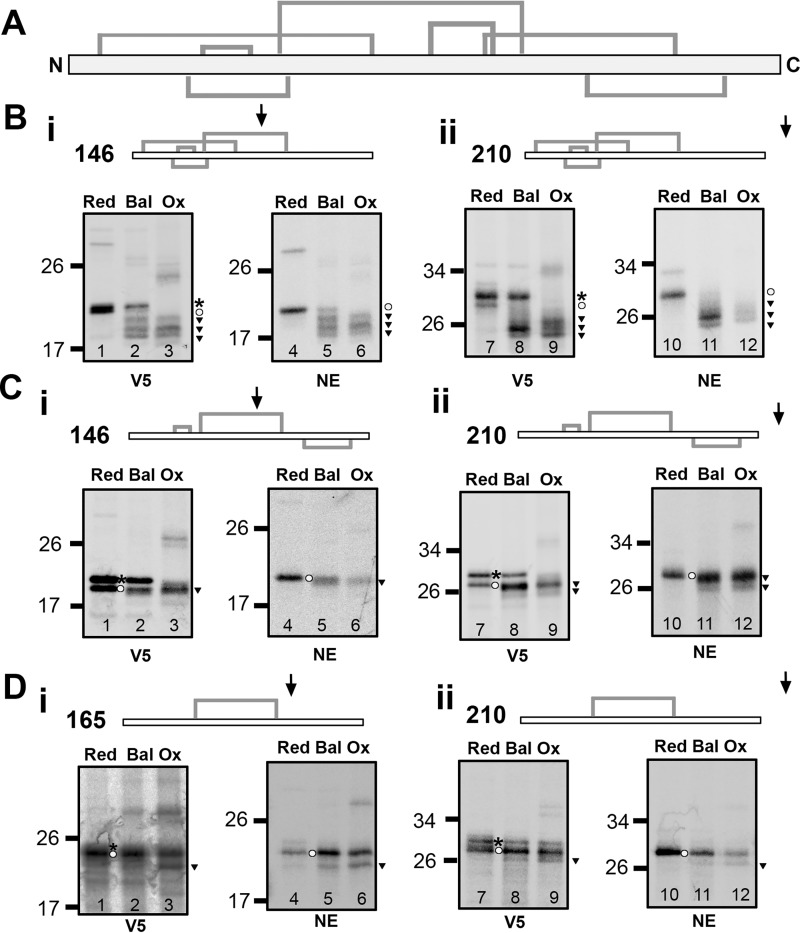
**Stochastic disulfide formation occurs even when the cysteine density decreases.** *A*, topology diagram showing the location of disulfides in the WT sequence of the disintegrin domain. *B–D*, non-reducing SDS-PAGE showing disulfide formation in stalled intermediates translated under reducing (*Red*), redox-balanced (*Bal*), or oxidizing (*Ox*) conditions for either the N-term cluster (*B*) or triple (*C*) or single (*D*) constructs immunoisolated using either a V5 or NE antibody as indicated. For each case, partially ER-exposed (146 or 165) intermediates (*i*) are compared with fully exposed (210) intermediates (*ii*). Topology diagrams above each panel show the position of disulfides in the relevant construct, with an *arrow* indicating the degree of ER exposure expected. Each condition was repeated three times, and representative data are shown. Symbols indicate the gel position of reduced preprotein (*asterisks*), reduced mature protein (*circles*), and oxidized mature protein bands (*inverted triangles*).

Analysis of the partially translocated N-term cluster intermediates (Fig. 6*B*, *i*) showed similar gel band patterns as observed for the WT protein, with multiple species present under both balanced and oxidizing conditions (Fig. 6*B*, *i*, *lanes 2*, *3*, *5*, and *6*). This is not surprising, as the ER-exposed portion of the disintegrin domain is identical to that of the WT protein. Increasing the length of the intermediate (Fig. 6*B*, *ii*) resulted in a prominent fast-migrating species under redox-balanced conditions (Fig. 6*B*, *ii*, *lanes 8* and *11*). The transition from multiple species to a single species shows that a single configuration is favored even in the absence of the C-terminal cysteines. Synthesis of the 146 intermediate of the triple construct in redox-balanced lysate showed two bands representing preprotein and mature protein (Fig. 6*C*, *i*, *lane 2*). This result contrasts to the array of disulfide-bonded species observed for the non-mutated and N-term cluster constructs and reflects the loss of specific disulfide arrangements. The preprotein runs faster in oxidizing lysate (Fig. 6*C*, *i*, *lane 3*), revealing disulfide formation in untargeted material. The ER-localized protein had a subtle change in mobility (Fig. 6*C*, *i*, *inverted triangles*) when comparing balanced and oxidizing conditions (Fig. 6*C*, *i*, *lanes 2*, *3*, *5*, and *6*) with the reduced controls (Fig. 6*C*, *i*, *lanes 1* and *4*). This indicates short-range disulfide formation occurring prior to full translocation and exposure of the domain to the ER. A more prominent disulfide-bonded species was detected in redox-balanced and oxidized samples (Fig. 6*C*, *ii*, *lanes 8*, *9*, *11*, and *12*) upon full domain translocation, indicative of a long-range disulfide. In contrast to the non-mutated protein, the process was less efficient, with loss of cysteine residues compromising long-range disulfide formation.

We translated a longer intermediate (165) for the single disulfide construct to allow ER exposure of both cysteines. Even with just two cysteines, disulfide formation (Fig. 6*D*, *i*, *inverted triangle*) occurred in oxidizing and redox-balanced lysates (Fig. 6*D*, *i*, *lanes 2*, *3*, *5*, and *6*) prior to full domain translocation. On full translocation, the disulfide formed, albeit inefficiently (Fig. 6*D*, *ii*, *lanes 8*, *9*, *11*, and *12*).

These results show that disulfide formation can occur upon partial exposure of the disintegrin domain despite lowering the cysteine density. This suggests that disulfide formation remains quasi-stochastic despite the lower cysteine content. In each case, removal of cysteines results in a less efficient disulfide formation process, indicating that disulfides in the native sequence that form stochastically in the early stages of folding influence the formation of subsequent disulfides later in the process.

## Discussion

In this study, we used an *in vitro* translation system to assess nascent chain disulfide formation in three proteins with diverse structures and disulfide bond patterns: β2M, prolactin, and the disintegrin domain of ADAM10. Our results indicate that disulfide formation occurs via two mechanisms that depend on a protein's secondary structure. In substrates with regular secondary structure, conformational folding drives disulfide formation. In contrast, in substrates with atypical secondary structure, folding of disulfide-rich domains occurs through a disulfide-driven process.

For β2M, disulfide formation depends on the protein's folding domain being fully exposed to the ER lumen; for prolactin, formation of the long-range disulfide requires that the protein is released from the ribosome–Sec complex. In both cases, there is a delay in disulfide formation despite exposure of multiple cysteines to the ER lumen. This absence of early cysteine coupling favors the structured precursor mechanism of folding. For β2M, it has already been established that disulfide formation follows partial folding (7); this study provided independent evidence via proteolysis assays that β2M undergoes folding before the disulfide forms, with an initial collapse of the nascent chain occurring during ER entry. In this case, partial folding of early intermediates is likely to spatially separate cysteines and prevent disulfide formation despite favorable oxidizing conditions. For prolactin, disulfide formation depends on the nascent chain's release from the ribosome–Sec complex, which suggests that tethering of the polypeptide to the ribosome prevents folding from reaching completion. Viable explanations for this dependence on release include a requirement for C- and N-terminal interactions to initiate the folding process (26, 27) and inhibitory interactions with cellular factors (28, 29) that are alleviated upon release. Although ribosome tethering clearly prevents conformational folding from being completed, the absence of cysteine coupling indicates that the translation intermediates are unlikely to be unstructured. Instead, we propose a mechanism similar to that identified for β2M, in which partially folded precursors spatially separate cysteines and prevent premature disulfide formation. These findings fit with those of other studies that have demonstrated partial folding at the cotranslational stage (30).

In the ER, the process of disulfide formation occurs via disulfide exchange with oxidoreductases. Access of these enzymes to buried disulfides in the core of the folded protein is unlikely. Considering the evidence of some folding occurring preceding disulfide formation for both β2M and prolactin, how does this model fit with the access requirements of catalyzing factors? To address this, we calculated solvent accessibility values from relevant 3D structures for each cysteine that forms a disulfide (31) to indicate the access folding factors would have to each cysteine pair in the folded state. For β2M, the disulfide is completely inaccessible to solvent (Fig. S4 and Table S4); for prolactin, one of the cysteines that makes up the long-range disulfide is buried, and the other is partially buried. In both cases, deep burial of one or more of the cysteines in each disulfide pair indicates that the relevant enzymes catalyze disulfide formation before the final fold is complete. Coupling this to our finding that folding occurs before disulfide formation indicates that positioning of cysteines for pairing occurs in a partially folded intermediate without restricting access to the relevant enzyme. This fits with other studies that found that PDI catalyzes disulfide formation at a late stage of folding (6).

In contrast to β2M and prolactin, disulfide formation occurs in disintegrin intermediates prior to the folding domain's full exposure to the ER lumen and results in a mixed population of non-native, disulfide-bonded species. A combination of the structural restrictions on the polypeptide when it is partially ER-exposed, combined with a stochastic mechanism of cysteine coupling, explains the array of species we observe. This is consistent with the quasi-stochastic model of folding, where disulfides form first in an unstructured precursor before conformational folding (4). Despite this stochastic mechanism, formation of a single disulfide-bonded form upon full domain exposure reveals cooperativity of the process. We conclude that the disintegrin domain has a disulfide-driven mechanism of folding, where quasi-stochastic cysteine coupling in the early stages provides the structural constraints for specific cysteine coupling at the later stages. It is important to acknowledge that use of stalled constructs may provide time for stochastic disulfide formation to take place. Such stalling would not occur during translation *in cellulo*. Future studies will investigate folding of the disintegrin domain in real time to understand whether such processes take place in a cellular context. In terms of access for oxidoreductases, the majority of cysteines are buried in the final fold (Fig. S4). This fits with the finding that disulfide formation and rearrangements take place in unstructured intermediates, with correct disulfides becoming buried and protected from further isomerization as a stable fold is achieved.

In a previous study, we identified ADAM10 as a substrate of the reductase ERp57, along with other proteins that share densely disulfide-bonded domains with low levels of secondary structure (13). This association is evidence of non-native disulfide formation and suggests that proteins with these structural features follow a stochastic cysteine-coupling process. Further examples of proteins that form non-native disulfides during folding include low-density lipoprotein receptor (10) and Bowman-Birk inhibitor (32), both of which contain disulfide-rich, atypically structured domains. The disintegrin constructs we investigated with lower cysteine density retained the features of the quasi-stochastic process. This suggests that secondary structure rather than the cysteine density is the deciding factor in the mechanism of cysteine coupling.

In this study, we assayed disulfide formation in stalled translation intermediates to provide insight into the folding mechanisms that achieve correct cysteine coupling in native protein structures. Our results provide novel insights into the relationship that exists between disulfide formation and protein folding. Our findings also raise questions for future studies to address, including whether these mechanisms apply to a broader number and diversity of substrates, how molecular chaperones and other ER factors interact with and influence the folding of nascent chains, and how folding events correlate with translation in real time. Such studies will help to further characterize the mechanisms described here and will advance our understanding of the fundamentally important process of oxidative protein folding in the cell.

## Experimental procedures

### Molecular graphics

The ribbon diagrams in Fig. 1 and Fig. S4 were drawn using the UCSF Chimera package, version 1.8, from the Resource for Biocomputing, Visualization, and Informatics at the University of California, San Francisco (33) using PDB codes 1A1M (β2M), 1RW5 (prolactin), and 6BE6 (ADAM10 disintegrin).

### Template generation

All protein constructs had the same C-terminal extension sequence with alternate N-terminal domains. This was either human β2M (extended β2M), bovine prolactin (extended prolactin), or human ADAM10 residues 456–550 (extended disintegrin). The extended β2M and extended prolactin constructs contained their native N-terminal signal sequences, whereas the extended disintegrin construct had the human-β2M signal sequence and the neo-epitope sequence (IIPVEEENP) added at the N terminus. These constructs were synthesized as plasmid DNA (Table S1) using GeneArt^TM^ gene synthesis (Thermo Fisher Scientific), with the sequence of each provided in Figs. S1–S3. Cysteine variants of the extended disintegrin construct were also synthesized in this way (Table S1). Mutations to remove glycosylation sites were introduced by site-directed mutagenesis using ACCUZYME^TM^ (Bioline). Plasmid DNA was used as a template for PCR using an appropriate forward primer that added a T7 promoter (Table S2) and various reverse primers that lacked stop codons (Table S3). PCR products were ethanol-precipitated, resuspended in nuclease-free water, and transcribed into RNA templates using T7 RNA polymerase (Promega) (37 °C, 2 h). The resulting RNA was ethanol-precipitated and resuspended in nuclease-free water for use as template in translation reactions. Templates containing stop codons for β2M and prolactin were generated as described previously (7, 14).

### Cell-free translation

Translations were performed using the Flexi® Rabbit Reticulocyte Lysate System (Promega) supplemented with 10 mm DTT (Melford) for reducing lysate, 5 mm G6Pi (Sigma) for redox-balanced lysate, or double-distilled H_2_O (oxidizing lysate). Typical translation reactions contained amino acids except methionine (20 μm), KCl (40 mm), EasyTag^TM^ EXPRESS^35^S Protein Labeling Mix (PerkinElmer Life Sciences, 1 μl/25-μl reaction), and RNA template. Semipermeabilized HT1080 human fibrosarcoma cells were prepared as described previously (20) and added to a concentration of ∼10^5^/25-μl translation reaction when required. Following assembly of components, translation reactions were incubated at 30 °C for 10 min for glycosylation experiments and all experiments with stalled β2M and prolactin intermediates or 30 min for templates with stop codons and stalled-disintegrin intermediates. After this time, samples were either treated with RNaseA (Sigma) (1 mg/ml, 5 min at 30 °C) or placed directly on ice. Samples were treated with *N*-ethylmaleimide (NEM; Sigma) at a concentration of 20 mm before further processing. Samples for glycosylation assays or downstream processing through ultracentrifugation were also treated with cycloheximide (2 mm). Alternative translation conditions were optimized for prolactin 30–310 (Fig. 3*E*, *ii*) with amino acids except cysteine replacing amino acids except methionine, and the KCl concentration was increased to 50 mm.

### Sample processing

Samples containing SP cells were centrifuged (16,000 × *g*, 30 s) following translation to isolate cell pellets, which were washed with 20 mm HEPES buffer pH 7.2, containing 110 mm KOAc, 2 mm MgOAc, and resuspended in SDS-PAGE sample buffer (20 μl) for direct SDS-PAGE analysis (crude samples) or in immunoprecipitation (IP) buffer (50 mm Tris (pH 7.5), 1% (v/v) Triton X-100, 150 mm NaCl, 2 mm EDTA, 0.5 mm PMSF, and 0.02% (w/v) sodium azide) for immunoisolation where indicated. Samples translated without SP cells were processed by various procedures following NEM treatment. In each case, either IP buffer (0.9 ml) was added for immunoisolation, the crude translation product was run directly on non-reducing SDS-PAGE, or ribosome–nascent chain complexes were isolated by ultracentrifugation. For the ultracentrifugation procedure, samples were loaded onto a sucrose cushion (2.5 m sucrose, 50 mm Tris (pH 7.5), 10 mm MgCl_2_, and 25 mm KCl) for 90 min at 265,000 × *g*. Pellets were rinsed with nuclease-free water and resuspended in nonreducing sample buffer for SDS-PAGE analysis.

### Immunoisolation procedure

Samples in IP buffer were incubated with 0.5% (v/v) protein A–Sepharose (PAS) (Generon) for 30 min (4 °C) and then centrifuged (2000 × *g*, 5 min) to clear samples of material that was bound nonspecifically. The resulting supernatant was incubated with PAS (0.5% v/v) and the indicated antibody at 4 °C overnight. The antibodies used were polyclonal rabbit anti-human β2M antibody (Dako, used at 1:1000), anti-V5 antibody (Invitrogen, used at 1:10,000) or an amino antibody to the mature form of placental alkaline phosphatase (used at 1:500) (34), which we termed the NE antibody. For NE immunoisolation procedures, PAS was pretreated with 1% BSA. Following overnight incubation with the antibody, samples were isolated by centrifugation (2000 × *g*, 5 min) and washed three times with 1 ml of IP buffer and once with 100 μl of 20 mm HEPES (pH 7.5). Protein was eluted by boiling the beads in non-reducing SDS-PAGE sample buffer. When samples were to be run under reducing conditions, 10 mm DTT was added to the sample before boiling, and when cooled, NEM was added (1 μl of 1 m NEM) before loading. Samples were run on SDS-PAGE, and the gels were fixed, dried, and exposed to either BioMax MR film (Kodak) for analysis by autoradiography or to phosphorimaging plates for analysis on a FLA-7000 Bioimager (Fujifilm).

### G6Pi recovery and auranofin treatment

Following translation of the 146 disintegrin intermediate (10 min at 30 °C) in oxidizing lysate, G6Pi was added (5 mm), and samples were incubated at 30 °C for 10 min. Aliquots were removed at the specified time points and treated with NEM for subsequent processing using V5 and NE immunoisolation, as described above. For the auranofin treatment, 10-min translations were performed before auranofin (Sigma) was added to relevant samples at a concentration of 20 μm. Following a further 10-min incubation at room temperature, 5 mm G6Pi was added for 30 min at 30 °C. Samples were treated with NEM before V5 immunoisolation and SDS-PAGE analysis, as described above.

## Author contributions

P. J. R. and N. J. B. conceptualization; P. J. R. and S. K. formal analysis; P. J. R., S. K., and X. C. investigation; P. J. R. writing-original draft; N. J. B. supervision; N. J. B. funding acquisition; N. J. B. writing-review and editing.

## Supplementary Material

Supporting Information

## Footnotes

3 The abbreviations used are:

ER: endoplasmic reticulum
PDI: protein disulfide isomerase
RNC: ribosome–nascent chain complex
SP: semipermeabilized
NEM: *N*-ethylmaleimide
aa: amino acid
NE: neo-epitope
IP: immunoprecipitation
G6P: glucose 6-phosphate
PAS: protein A-Sepharose.
