# Supplementary material for: Protein secondary structure determines the temporal relationship between folding and disulfide formation
Source: J Biol Chem. 2020 Jan 17;295(8):2438–48. doi: 10.1074/jbc.RA119.011983 (PMC7039548; doi:10.1074/jbc.RA119.011983)
Supplement: Supporting Information [file supp_295_8_2438__index.html]

Protein secondary structure determines the temporal relationship between folding and disulfide formation — Protein structure determines when disulfides form — Protein secondary structure determines the temporal relationship between folding and disulfide formation — Protein structure determines when disulfides form — Supporting Information 

# Protein secondary structure determines the temporal relationship between folding and disulfide formation

## Supporting Information

- Supporting Information - Additional details of reagents used
