# Supplementary material for: Protein secondary structure determines the temporal relationship between folding and disulfide formation
Source: J Biol Chem. 2020 Jan 17;295(8):2438–48. doi: 10.1074/jbc.RA119.011983 (PMC7039548; doi:10.1074/jbc.RA119.011983)
Supplement: Supporting Information [file supp_RA119.011983_157241_2_supp_458736_q45lk5.docx]

Protein secondary structure determines the temporal relationship between folding and disulfide formation

Philip J Robinson, Shingo Kanemura, Xiaofei Cao, Neil J Bulleid

Table S1: Plasmid list

Table S2: Forward primers for transcription/translation template generation

Table S3: Reverse PCR primers to generate templates for stalled intermediates

Figure S1. DNA sequence of the extended β2M construct

Figure S2: DNA sequence of the extended prolactin construct

Figure S3: DNA sequence of the extended disintegrin construct

Table S4: Solvent accessibility of each cysteine residue involved in disulfide bonding

Figure S4. The solvent accessibility of disulfide bonds in the native structure of each substrate

**Table S1: Plasmid list**

| **Plasmid** | **Description** |
| --- | --- |
| Extended-β2M | DNA Sequence in Fig.S1 |
| Extended- β2M AST | Extended- β2M with N120A mutation |
| Extended-prolactin | DNA Sequence in Fig.S2 |
| Extended-prolactin AST | Extended-prolactin with N230A mutation |
| Extended-disintegrin | DNA Sequence in Fig.S3 |
| Extended-disintegrin AST | Extended-disintegrin with N125A mutation |
| Extended-disintegrin AST: N-term cluster | Extended-disintegrin with N125A mutation and cysteines at positions 77,84,85,98,104,110 and 117 mutated to serines. |
| Extended-disintegrin AST: triple | Extended-disintegrin with N125A mutation and cysteines at positions 34, 45, 58 , 69 , 77 ,84 , 85, 104 and 110 mutated to serines. |
| Extended-disintegrin AST: single | Extended-disintegrin with N125A mutation and cysteines at positions 34, 45, 47, 53, 58, 69, 77, 84, 85, 98, 104, 110, 117 mutated to serines |

**Table S2: Forward primers for transcription/translation template generation**

| Extended-β2M Fwd* | GATGGCTAATACGACTCACTATAGGGTCAGGCCACCATGAGCAGATCTGTGGCCCTGG |
| --- | --- |
| Extended-β2M mature Fwd | GATGGCTAATACGACTCACTATAGGGTCAGGCCACCATGATCCAGCGGACCCCCAAGATCCAGGTGTA |
| Extended-prolactin Fwd | GATGGCTAATACGACTCACTATAGGGTCAGGCCACCATGGACAGCAAAGGTTCGTCGC |
| Extended-prolactin mature Fwd | GATGGCTAATACGACTCACTATAGGGTCAGGCCACCATGACCCCCGTCTGTCCCAATGGGC |

*This primer is also used to generate disintegrin templates as the extended-disintegrin construct contains the β2M signal sequence.

**Table S3: Reverse PCR primers to generate templates for stalled intermediates**

| Length of intermediate from start codon (aa) | | |  |
| --- | --- | --- | --- |
| β2M | Prolactin | Disintegrin | Primer sequence |
| 105 | - | - | CACGTGGTTCACTCTGCAG |
| 141 | - | 146 | CATCATCATCATCATGGTGCTGTC |
| - | - | 155 | AGCAGAGCTGGCGCT |
| - | - | 165 | AGAGCTGGCTGTGGCTC |
| 165 | 275 | - | GCCGCCAGCAGATGTAGAG |
| 175 | 285 | 180 | TGTAGATCCGCCTGTAGAGC |
| - | 300 | 195 | AGCTCCGCCAGTAGATCCT |
| - | 305 | 200 | TGTTCCAGTAGAGGCAGCTC |
| - | 310 | 205 | TCCGCCCCCAGCAGCT |
| - | - | 210 | AGAAGAAGCTCCGCCTCC |
| - | 330 | 225 | TGTTGTTCCGGTGCCTGTAG |

**Figure S1. DNA sequence of the extended β2M construct**

ATGAGCAGATCTGTGGCCCTGGCTGTGCTGGCCCTGCTGTCTCTGTCTGGCCTGGAAGCCATCCAGCGGACCCCCAAGATCCAGGTGTACAGCAGACACCCCGCCGAGAACGGCAAGAGCAACTTCCTGAACTGCTACGTGTCCGGCTTCCACCCCAGCGACATCGAGGTGGACCTGCTGAAGAACGGCGAGCGGATCGAGAAGGTGGAACACAGCGACCTGAGCTTCAGCAAGGACTGGTCCTTCTACCTGCTGTACTACACCGAGTTCACCCCCACCGAGAAGGACGAGTACGCCTGCAGAGTGAACCACGTGACCCTGAGCCAGCCCAAGATCGTGAAGTGGGACCGGGACATGAACAGCACCGGCAAGCCCATCCCCAACCCTCTGCTGGGCCTGGACAGCACCATGATGATGATGATGTCCGGCACCGCCAGCGCCAGCTCTGCTGGATCTGGCGGCGGAGCCACAGCCAGCTCTACATCTGCTGGCGGCACAAGCACCGGCTCTACAGGCGGATCTACAGCAGGCGCTGCTGGCGCAACAGGCGGAGGATCTACTGGCGGAGCTGCCTCTACTGGAACAGCTGCTGGGGGCGGAGGCGGAGCTTCTTCTGGAACAGGCACAGGCGCCAGCGGCGCTACAGGCACCGGAACAACA

**Figure S2. DNA sequence of the extended prolactin construct**

ATGGACAGCAAAGGTTCGTCGCAGAAAGGGTCCCGCCTGCTCCTGCTGCTGGTGGTGTCAAATCTACTCTTGTGCCAGGGTGTGGTCTCCACCCCCGTCTGTCCCAATGGGCCTGGCAACTGCCAGGTATCCCTTCGAGACCTGTTTGACCGGGCAGTCATGGTGTCCCACTACATCCATGACCTCTCCTCGGAAATGTTCAACGAATTTGATAAACGGTATGCCCAGGGCAAAGGGTTCATTACCATGGCCCTCAACAGCTGCCATACCTCCTCCCTTCCTACCCCGGAAGATAAAGAACAAGCCCAACAGACCCATCATGAAGTCCTTATGAGCTTGATTCTTGGGTTGCTGCGCTCCTGGAATGACCCTCTGTATCACCTAGTCACCGAGGTACGGGGTATGAAAGGAGCCCCAGATGCTATCCTATCGAGGGCCATAGAGATTGAGGAAGAAAACAAACGACTTCTGGAAGGCATGGAGATGATATTTGGCCAGGTTATTCCTGGAGCCAAAGAGACTGAGCCCTACCCTGTGTGGTCAGGACTCCCGTCCCTGCAAACTAAGGATGAAGATGCACGTTATTCTGCTTTTTATAACCTGCTCCACTGCCTGCGCAGGGATTCAAGCAAGATTGACACTTACCTTAAGCTCCTGAATTGCAGAATCATCTACAACAACAACTGCAACAGCACCGGCAAGCCCATCCCCAACCCTCTGCTGGGCCTGGACAGCACCATGATGATGATGATGTCCGGCACCGCCAGCGCCAGCTCTGCTGGATCTGGCGGCGGAGCCACAGCCAGCTCTACATCTGCTGGCGGCACAAGCACCGGCTCTACAGGCGGATCTACAGCAGGCGCTGCTGGCGCAACAGGCGGAGGATCTACTGGCGGAGCTGCCTCTACTGGAACAGCTGCTGGGGGCGGAGGCGGAGCTTCTTCTGGAACAGGCACAGGCGCCAGCGGCGCTACAGGCACCGGAACAACA

**Figure S3. DNA sequence of the extended disintegrin construct**

ATGAGCAGATCTGTGGCCCTGGCTGTGCTGGCCCTGCTGTCTCTGTCTGGCCTGGAAGCCATTATTCCGGTGGAAGAAGAAAACCCGGGGCAGCCTATCTGCGGCAACGGAATGGTGGAACAGGGCGAAGAGTGCGACTGTGGCTACAGCGACCAGTGCAAGGACGAGTGCTGCTTCGATGCCAACCAGCCTGAGGGCAGAAAGTGCAAGCTGAAGCCTGGCAAGCAGTGCAGCCCTAGCCAGGGACCTTGTTGCACAGCCCAGTGTGCCTTCAAGAGCAAGAGCGAGAAGTGCCGCGACGACTCTGATTGTGCTAGAGAGGGCATCTGCAACGGCTTCACCGCTCTGTGTCCTGCCAGCGATCCTAAACCTAACAGCACCGGCAAGCCCATCCCCAACCCTCTGCTGGGCCTGGACAGCACCATGATGATGATGATGTCCGGCACCGCCAGCGCCAGCTCTGCTGGATCTGGCGGCGGAGCCACAGCCAGCTCTACATCTGCTGGCGGCACAAGCACCGGCTCTACAGGCGGATCTACAGCAGGCGCTGCTGGCGCAACAGGCGGAGGATCTACTGGCGGAGCTGCCTCTACTGGAACAGCTGCTGGGGGCGGAGGCGGAGCTTCTTCTGGAACAGGCACAGGCGCCAGCGGCGCTACAGGCACCGGAACAACA

**Table S4: Solvent accessibility of each cysteine residue involved in disulfide bonding**

|  | Cys 1 residue number | Cys1 solvent accessibility ~Å² | Cys2 residue number | Cys2 solvent accessibility, ~Å² |
| --- | --- | --- | --- | --- |
| β2M | 45 | 0 | 100 | 0 |
| Prolactin | 34 | 36.15 ± 9.05 | 41 | 43.75 ± 13.87 |
|  | 88 | 25.35 ± 5.81 | 204 | 2.7 ± 3.64 |
|  | 221 | 15.15 ± 9.46 | 229 | 91.15 ± 11.59 |
| Disintegrin | 34 | 23 | 69 | 5 |
|  | 45 | 0 | 58 | 1 |
|  | 47 | 14 | 53 | 24 |
|  | 57 | 5 | 89 | 5 |
|  | 77 | 0 | 85 | 7 |
|  | 84 | 1 | 110 | 7 |
|  | 98 | 28 | 117 | 25 |

Solvent accessibility is determined using disulfide bond analysis software and the PDB files 1A1M, 1RW5 and 6BE6: <http://149.171.101.136/python/disulfideanalysis/index.html> The values for prolactin are an average from the NMR structure with standard deviations shown.


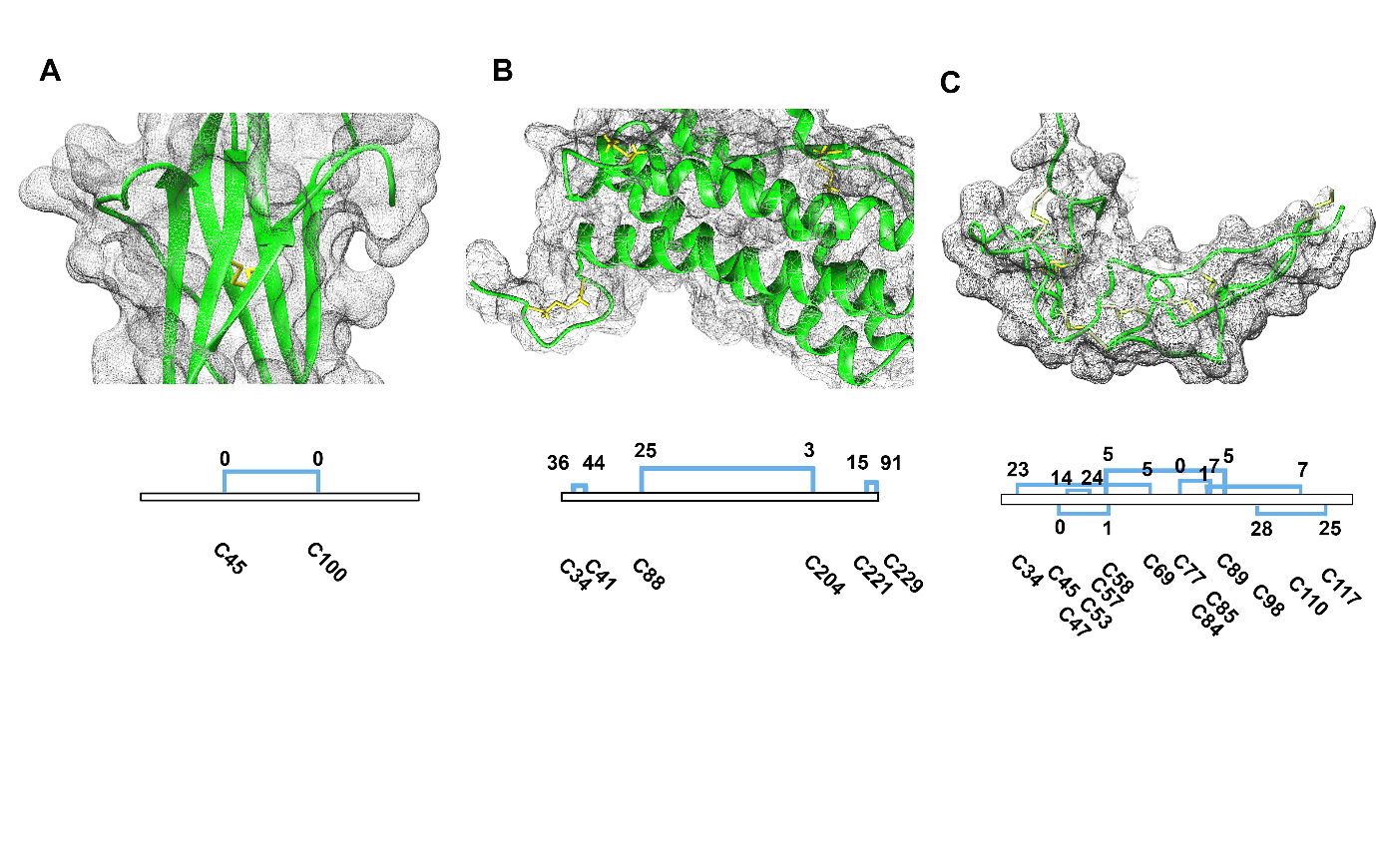


**Figure S4. The solvent accessibility of disulfide bonds in the native structure of each substrate** Ribbon diagrams (green) of (A) β2M (PDB 1A1M), (B) Prolactin (PDB 1RW5) and (C) the disintegrin domain of ADAM10 (PDB 6BE6) with disulfide bonds highlighted (yellow) and the surface represented as a transparent mesh. Below each structure is a topology diagrams displaying the location of each cysteine residue that makes up a disulfide bond. Cysteine numbering is shown below each diagram and solvent accessibility values are shown above or below the relevant cysteine in the diagram as taken from Table S4.
